# Does Usage of Online Social Media Help Users With Depressed Symptoms Improve Their Mental Health? Empirical Evidence From an Online Depression Community

**DOI:** 10.3389/fpubh.2020.581088

**Published:** 2021-01-25

**Authors:** Yingjie Lu, Taotao Pan, Jingfang Liu, Jun Wu

**Affiliations:** ^1^School of Economics and Management, Beijing University of Chemical Technology, Beijing, China; ^2^School of Management, Shanghai University, Shanghai, China

**Keywords:** online depression community, sense of belonging, sense of support, sense of identity, trust, informational support, emotional support, socializing support

## Abstract

Online depression communities offer people with depressed symptoms new opportunities to obtain health information and provide social support for each other to fight against the depression. We sought to investigate whether usage of online community help improve depression outcomes and determine which types of usage behaviors have positive or negative effects on depression. We proposed that two dimensions of the sense of belonging (sense of identity and trust) and three dimensions of the sense of support (informational, emotional, and socializing) have significant effects on depression, and further considered gender difference and its effect on depression. We obtained a dataset consisting of 465,337 posts from 244 members from a popular online depression community to test all 10 proposed hypotheses. The results reveal that (i) the sense of shared identity, trust, informational support, and emotional support have positive effects on depression, while socializing support have negative effects on depression, and (ii) the sense of shared identity and trust have more positive effects on depression for female users than male users while socializing support has a more negative effect on depression for female users than for male users. The findings have important practical implications for designers and managers of online depression communities.

## Introduction

Depression is considered to be associated with a range of negative outcomes, including cognitive impairment, substance abuse, self-harm and suicide (1). A survey from WHO pointed out that more than 300 million patients are suffering from depression and the population increased by 18% in the past 10 years (2). Despite the prevalence of depression, those with depressed symptoms are frequently undiagnosed, as they do not perceive a need for help and do not seek clinical services (3, 4). There are also some other barriers that prevent them from seeking clinical services, including the lack of necessary knowledge about diagnosis and treatment, privacy concerns, and so on.

It has been a hot issue that how to treat depression. Social support, especially peer support, is widely considered to be protective against depression (5). However, those suffering from depression have a severe fear of disclosing their mental illness and seeking help from others due to the stigma and discrimination associated with depression (6), which, in turn, prevents interactions with others and increases their feelings of social isolation.

In recent years, social media is becoming popular as a communication tool for information sharing, searching and communicating (7). Patients are turning to social media for health information and communication. Some healthcare social media platforms, such as online depression communities are being used by people with depressed symptoms to obtain health information and provide social support for each other to fight against the depression. Therefore, online depression communities have become increasingly recognized as promising platforms for communication about depression (8).

One stream of research suggests that online interactions with fellow users can improve anxiety (9, 10), tension, stress, and various other negative mood states (11) and help alleviate depressive symptoms. However, while these studies focus on the benefits of online depression communities, some scholars express the opposite opinion that long-term online interactions with major patients may worsen the symptoms of depression. According to emotional contagion theory, individuals' emotional states easily spread across socially connected individuals in a social network. While some users show positive emotions to encourage each other to overcome their diseases, others regard online communities as a channel to vent negative emotions. Negative sentiment has been shown in some studies to be highly prevalent and more common than positive sentiment in mental health communities, especially in online depression communities (12). Thus, members of depression communities who are exposed to other members' negative emotions are likely to show significant increases in anxiety, anger, and other negative emotions, even the risk of suicide. These contradictory conclusions may result in difficulties in evaluating the value of online interactions for users. Therefore, it is still an issue of debate concerning whether online interaction is beneficial or harmful to users with depressed symptoms and, if so, to what extent.

While many studies have argued about the positive or negative effects of social media use on the users with depressed symptoms, few of them investigate which factors are associated with positive or negative mood change. Previous research has mainly focused on the content analysis of posts by users with depressed symptoms. Some scholars adopted topic modeling techniques to extract hot topics of interest to users (13), and then analyze the evolution of topics over time to judge whether the users' depressive symptoms have been improved. Some scholars used well-known sentiment lexicons to extract sentiment terms from the messages and calculate their sentiment scores to judge emotional changes (14). However, these analytical methods used in previous studies place limitations on describing and understanding the mechanisms of how online community participation is related to the improvements on depression symptoms. Users involved in online depression communities have different usage behaviors due to different motivations and intentions, and achieve different social and psychological benefits to satisfy their diverse needs, which may have a complicated influence on their psychological health and well-being. Therefore, further analysis would be necessary to determine how different usage behaviors of online social media have different impact on the psychological state.

In addition, some recent studies highlight gender difference in online health communities (15). They found that there is a significant difference in the demand for online health communities between male and female users (16), resulting in their different usage behaviors. Hence, in this study, we are also concerned with the issue of gender difference and examine whether gender difference can mediate the effect of online community participation on depression improvements. Thus, in our study, we try to investigate the following research questions:

RQ1. Can usage of online social media help improve depression outcomes?
RQ2. Which types of usage behaviors have positive (a) or negative (b) effect on depression?
RQ3. Are there differences concerning the effect of online community participation on depression for male and female users?

## Theoretical Background and Research Hypothese

### Sense of Online Community

Some previous studies applied social psychology to virtual communities and proposed that user participation in online communities aims mainly to develop a sense of community with others who share similar conditions or concerns (17). Sense of community is a feeling that members have of belonging, a feeling that members matter to one another and to the group, and a shared faith that members' needs will be met through their commitment to be together (18). Previous studies suggested that sense of community can help diminish the feelings of isolation and lack of support, buffering the negative impact of stress, depression, and anxiety on health, and promote positive psychosocial outcomes (19). Sense of community involves two critical aspects: the sense of belonging and the sense of support. Therefore, in this study, we propose that motivation for users with depressed symptoms participation in virtual communities may be a desire to develop a sense of belonging to depression communities and a sense of support from other depression members.

#### The Sense of Belonging

The sense of belonging involves the feeling, belief, and expectation that one fits in the group and has a place there, a feeling of acceptance by the group, and a willingness to sacrifice for the group. The sense of belonging leads members to identify with the group (20), trust group members (21), and feel morally obliged to participate in the group activities. We focus on two key facets of the sense of belonging: the sense of shared identity and trust, both of which have been found in some social psychological theories to better understand the sense of belonging and its effect on psychological well-being.

According to social identity theory, individuals who have a strong sense of identity with the group define themselves in terms of their group membership and describe themselves as being in line with the stereotype of the group (22). They feel morally obliged to help others within the organization on the basis of shared membership (23). In online depression communities, users view themselves as members of the community and consider it a duty to assist other users in improving depression treatment and outcomes (24). When they think they have the ability to help others, they will feel very happy. Therefore, we propose the following hypothesis:

*H1: The sense of shared identity has a positive effect on depression in online depression communities*.

In addition to a sense of shared identity, trust is also an important factor that positively affects a member's sense of belonging to the virtual community. Many studies suggested that trust can help group members understand how to avoid possible misunderstandings in their communications with other members and how to reduce the possibility of opportunistic behavior (25). When individuals trust that their shared knowledge will be used appropriately, they are more willing to engage in online interactions, interact with community members, and create and maintain interpersonal relationships with their peers (26). In online depression communities, trust is an important determinant of online interaction. We can postulate that those users who a high level of trust are more willing to engage in online communities to freely exchange information and opinions, without worrying that shared knowledge and personal information could be misused or abused. In conclusion, we think that trust can create a favorable atmosphere of free discussion and expression about depression, which helps users improve their depression conditions. Therefore, we propose the following hypothesis:

*H2: The users with a high level of trust* are *more likely to improve depression outcomes in online depression communities*.

#### The Sense of Support

The sense of support is the feeling that members' needs will be met by the resources received through their membership in the group. In recent years, the associations between social support and depressive symptoms have been widely documented in many studies. Some researchers adopted uses and gratifications (U&G) theory to explain what social and psychological needs motivate individuals to participate in social media (27). According to U&G studies, there exist three major types of needs that can be gratified by using social media: informational, emotional and socializing (28, 29). Therefore, we suggest that the sense of support received from online depression communities could be divided into three types: informational support, emotional support and socializing support, which satisfy different needs of users and have important effects on their depressive conditions.

Many users affected by depressed symptoms hoped to obtain useful medical knowledge to help themselves manage their depression conditions or find some valuable diagnosis and treatment information about depression from the experiences shared by other members through online depression communities (30). Social media provide new channels for the users seeking informational support to communicate information or knowledge on the topics of interest. These information and knowledge help users learn to better cope with psychological stress, eliminate some negative feeling of fears and anxiety, and make them more confident and involved in their healthcare processes, which would help them improve depressive symptoms. Therefore, we propose the following hypothesis:

*H3: Informational support has a* positive *effect on depression in online depression communities*.

Health care social media is not only used to exchange health information (31–33), but also provides a platform for emotional communication (34–36), where they can vent their feeling and receive emotional support from group members. For many users affected by depressed symptoms suffering long-term psychosocial problems, including fear of treatment, loss of work role, dependency and alienation, they regard social media as a good platform to relieve stress and hope to receive emotional support from fellow users (37). They expressed their sympathy and pity for other members and encourage each other to fight their mental illness (38). We believe that emotional expressions, such as sending positive energy, showing compassion and empathy, are important for users to improve their psychosocial well-being. Therefore, we propose the following hypothesis:

*H4: Emotional support has a positive effect on* depression *in online depression communities*.

Online communities give people additional opportunities to make new friends. Many users affected by depressed symptoms regard online communities as important interpersonal communication channels to create and maintain social ties with their peers. Socializing support received from online communities could bring them some psychological benefits. On the one hand, social ties in online communities are considered by users affected by depressed symptoms as an important source of companionship, which make them feel that there are others who enjoy their presence (39) and they are not isolated from the world. Social companionship that users received from online communities could lead to higher levels of psychological well-being. On the other hand, many users expect to receive attention when they participate in online communities. They feel appreciated and respected if their online social activities attract much attention (40). The feeling of being appreciated, loved, and understood will bring them higher levels of psychological well-being. Therefore, we propose the following hypothesis:

*H5: Socializing support has a positive* effect *on depression in online depression communities*.

### Gender Difference

Gender difference on depression have been documented for many years and thought to be an important determinant of treatment outcome. A consistent finding in the depression literature is that women are about twice as likely as men to be diagnosed with depressive symptoms (41–45). There have been many possible explanations for this gender disparity on depression, including biological factors (genetic vulnerability and hormonal influences), social factors (social role and status, social support), and psychological factors (coping style and neuroticism) (46–52).

In this study, we focus on gender difference in online health communities and explore whether there are different effects of online community participation on depressive symptoms for male and female users. According to the existing theories research on gender difference on depression, we proposed that there are two possible factors that could explain the gender difference in online community participation and its effect on the development of depression. On the one hand, we considered some gender difference in psychological factors, such as the coping style on depressive (emotion-focused coping or problem-focused coping), could play an important role in influencing users' motivation and behavior in online communities. On the other hand, some social factors that may affect depression, such as social support, are found to be closely related to the use of online communities. Since it is widely recognized that there are significant gender difference in the perception and utilization of social support (53, 54), we can speculate that different types of social support received from online community members may have different effects on depression for men and women users. Based on the arguments above, we propose some hypotheses suggesting that gender may play a key moderating role in the relationship between online community participation and depression.

#### Gender Difference in the Sense of Belonging

There is a popular belief that women are more vulnerable than men to the negative effects of undesirable life events. Accordingly, women have a stronger affiliative style than men, because they feel more in need for support in managing their psychological health (55). We believe that, in the context of online depression communities, women would be more attached to online communities and feel a stronger sense of shared identity with other members than men. In addition, many studies report differences in how women and men cope with stress. Men tend to deal with stress by problem-focused coping, while women tend to deal with stress by emotion-focused coping (56, 57). Online interaction can make women perceive the sense of having a supportive and understanding person to talk to in times of trouble. Therefore, compared to men, women are more likely to identify with online communities and more engaged in online activities. These distracting activities will interrupt the effects of depressed mood on thinking, relieve their distress and improve their psychological well-being. Therefore, we propose the following hypothesis:

*H6-a: The positive influence of the sense of shared identity on depression is stronger for women than men in online depression communities*.

Social role theory is another possible explanation for such gender difference in online interactions, suggesting that the core of gender difference between men and women is that men are more agentic and women are more communal (58). Compared to men, the communal nature of women and their open cooperativeness leads us to believe that some social capital constructs of social ties, such as trust, have a deeper and more meaningful impact on the relationships of women within online communities (59). We believe that women will place a stronger emphasis on trust because trust as a facilitating factor for sustaining social relationships will have a stronger influence on women than men. In the meanwhile, high levels of trust make women likely to express their feelings to others, resulting in more self-disclosure and a decrease in feelings of social isolation and loneliness, which make women benefit more than men from online interactions. Therefore, we propose the following hypothesis:

*H6-b: The positive influence of trust on depression* is *stronger for women than men in online depression communities*.

#### Gender Difference in the Sense of Support

According to the response styles theory, there are different coping styles utilized by men and women in response to depression: problem-focused coping and emotion-focused coping (60), which make users receive different types of support to improve their psychological well-being.

Researchers suggested that men tend to use problem-focused coping to solve concrete problems (57). Online community is viewed more as an important health communication channel for men than for women, so male users are more likely to exhibit information seeking behaviors, such as searching valuable knowledge about depression or browsing some topics related to depression treatment. They could gain much valuable work-related information and previous experiences of other users through online platforms to come up with solutions to mental problems they encountered in daily life and thus reduce levels of depression. Therefore, we propose the following hypothesis:

*H6-c: Informational support has a* more *positive effect on depression for men than women in online depression communities*.

Women are considered more likely to use emotion-focused coping to obtain emotional support (56). Online community is viewed more as an emotion-exchange platform for women than for men, on which women lacking social support could seek emotional support from their peers. The emotional support in online depression communities involves the provision of caring, empathy and love to other members. Considering that women have lower self-concept and self- reliance than men, emotional support can bring more psychological benefits to women users, such as reduced loneliness, lowered anxiety, and increased feelings of well-being. Therefore, we propose the following hypothesis:

*H6-d: Emotional support has a more positive effect on depression for women than men in online depression communities*.

In addition to the above two types of supports, users could benefit from socializing support during long-term involvement in online communities in many other ways. Some researches revealed that women will experience more social isolation and exclusion as they are less likely to have access to socializing support (61), and thereby are more likely to engage in intimate and self-disclosing relationships than men (62, 63). Consequently, female users are expected to be more willing to use online communities to build their own social networks and develop intimate relationships with other members. We believe that compared to male users, female users can gain more psychological benefits from socializing support received in online communities, such as eliminating their fear about disclosing their status to their family members and other significant members of their social networks, reducing their social isolation and loneliness and promoting their well-being. Therefore, we propose the following hypothesis:

*H6-e: Socializing support has a more positive effect on depression for women than men in online depression communities*.

## Methods

### Research Context and Data Collection

We chose a Chinese online depression community as the data source. The community name is “Sunshine Project” (www.sunofus.org). It is a non-profit website and DeTao Global Innovation Network Co., Ltd. provides free server and network technical support for the website. The online forum was classified into 15 discussion boards where users could talk about all kinds of topics related to depression. The moderators are from senior members. According to forum management regulations, senior members who have registered for more than 3 months, have a total online time of more than 50 h and posted more than 100, can apply for moderators. All members can express their personal opinions and vote on the applicants, and finally the applicants are approved by the forum administrator. The online community is one of the most popular online healthcare platforms for Chinese people with depressed symptoms. On the website, users can talk about their depression symptoms, seek valuable healthcare information, and share their treatment experiences. In the meanwhile, the website is considered as an emotional support platform, allowing users to freely express their concerns and feelings, such as venting their emotional distress and showing sympathy and encouragement to peers, which are very important for them to alleviate depressive symptoms. However, according to forum management regulations, members are not allowed to post illegal posts. If an illegal post is reported, the moderators have the right to delete the illegal post or even prohibit the poster from posting or visiting the website.

We collected the empirical data from the website from January 1, 2006 to December 31, 2015 due to the availability of data. We found that there are 10,843 members who participated in the online community and published a total of 954,537 posts. Among them, 244 members posted more than 500 posts, including initial posts and replies to others' posts. These members were defined as active users, accounting for 2.25% of the total number. We only collected the information available to the public, such as the authors of the posts, post title and body, timestamp, and so on. The personal information is private and sensitive, and should not be included in our sample due to ethical or legal concerns. We therefore obtained a sample consisting of 465,337 posts from 244 active users.

We further made a content analysis of the posts and classified them into informational or emotional posts. First, we randomly selected 5,000 posts and manually annotated them as informational or emotional. After a small number of ambiguous or incomplete posts were excluded, we finally annotated 2,402 informational posts and 1,838 emotional posts from the training sample. Next, we extracted keywords, respectively, from the two types of posts by using TF-IDF method to construct information dictionary and sentiment dictionary. Finally, we used the dictionary-based classification method to obtain 304,541 informational posts and 111,523 emotional posts from the sample data.

### Method

Panel data regression analysis was employed in this study to explore which factors have positive or negative effects on depression. In our empirical model, depressed mood is used as the dependent variable and two types of the sense of belonging (the sense of identity, trust) and three types of the sense of support (informational support, emotional support and socializing support) are used as independent variables, as shown in Equation (1).

(1) $$\begin{array}{l} Mood_{i,t+1}=\beta_{0}+\beta_{1}Identity_{i,t}+\beta_{2}Trust_{i,t}+\beta_{3}Informational_{i,t}\\ \;+\beta_{4}Emotional_{i,t}+\beta {+}_{5}Socializing_{i,t}+\varepsilon_{i,t} \end{array}$$

Table 1 provides a summary description of all variables. Each period t refers to 1 day. Period t+1 is the day after period t. As the dependent variable in our empirical analysis, the severity of depressed symptoms was measured by using the sentiment score of written posts from online users. In our study, we did not assess the depression severity level of online users using standardized diagnostic instruments or structured clinical interviews. On one hand, there are different types of users with depressed symptoms participating in online depression communities. Some of them are clinically depressed patients, while the others are users with subclinical depressed mood. Many of them with self-reported depressive symptoms have not made the diagnosis of depression but they should also be included in our study. On the other hand, prior studies (64) pointed out that the established measures of depression have shown some psychometric limitations. Clinical theories of depression assume that depression is most strongly manifested in thoughts and feelings about the self (65), and thus depressive symptoms are hard to detect by outside observers. Instead, we consider that the assessment of users' depressed symptoms by analyzing the linguistic content of posts might be more appropriate for those online users with depressed symptoms than an independent assessment of clinical outcomes. These posts regarding thoughts and feelings about the self make depression more easily detectable. Numerous studies (66, 67) have demonstrated that depressed individuals show distinct linguistic patterns that parallel the symptoms of depression. It is widely recognized (68–70) that depressed individuals tend to use more negative emotive words, particularly those related to sadness, and fewer positive emotive words. These findings provide strong support for the use of sentiment scores of written posts to measure the depressed symptoms. We use the variable *Mood*_*i, t*+1_ to represent the sentiment scores of posts written by user i in period t+1.

**Table 1:** Summary description of all variables.

**Variables**	**Description**
**Dependent variable**	
Mood_i, t+1_	Sentiment scores of posts written by user i in period t+1
**Independent variables**	
Identity_i, t_	The number of initial posts written by user i in period t
Trust_i, t_	The number of replies to others' posts written by user i in period t
Informational_i, t_	The number of replies to informational posts received by user i from other users in period t
Emotional_i, t_	The number of replies to emotional posts received by user i from other users in period t
Socializing_i, t_	The number of users interacting with user i in period t
**Moderator variable**	
Gender	Dummy variable with 1 for male and 0 for female

In this study, we used an open platform for sentiment analysis developed by Baidu AI group to obtain sentiment scores of posts. In the Baidu AI platform, a pre-developed sentiment dictionary with sentiment words and their sentiment values were stored in the database. We can automatically calculate sentiment scores of Chinese text using Natural Language Processing (NLP) based on the sentiment dictionary. We briefly summarize the calculation steps as follows. Firstly, posting text is segmented into individual words including nouns, verbs, and adjectives, and then the frequencies of all the words are computed; secondly, each word will be assigned a sentiment value by matching the words with the sentiment score in the pre-developed database automatically; thirdly, sentiment score of posting text is obtained by multiplying the frequency of each word with the sentiment score and then summing them up. Some examples with sentiment scores were listed in the Appendix 1.

We used two independent variables to measure the two key facets of the sense of belonging: the sense of shared identity and trust. The sense of shared identity can be achieved through their active contributions to the online community. Thus, we used the variable *Identity*_*i, t*_, representing the number of initial posts written by user i in period t, to measure the sense of shared identity of user i. Trust is the willingness to engage in online discussions and interact with community members. Thus, we used the variable *Trust*_*i, t*_, representing the number of replies to others' posts written by user i in period t to measure the trust of user i.

We used three independent variables to measure three types of the sense of support. Informational support refers to the degree to which a user's need for information and knowledge about depression is gratified through interaction with others. We used the variable *Informational*_*i, t*_, representing the number of replies to informational posts received by user i from other users in period t, to measure perceived informational support for user i. Emotional support refers to the degree to which a user's emotional need is gratified through interaction with others. We used the variable *Emotional*_*i, t*_, representing the number of replies to emotional posts received by user i from other users in period t, to measure perceived emotional support for user i. Socializing support refers to the degree to which a user's need to maintain social ties with their peers is gratified through interaction with others. We used the variable *Socializing*_*i, t*_, representing the number of users interacting with user i in period t, to measure perceived socializing support for user i.

## Results

### Preliminary Analysis

Table 2 presents descriptive statistics of the dependent variable and independent variables measuring the sense of online community that were used in the study. On average, each user wrote 55.65 initial posts and 747.74 replies to others' posts. In the meanwhile, each user could receive an average of 27.80 replies to their informational posts and 3.36 replies to their emotional posts, and interact with 27.92 other members. We further divided the full sample into subsamples according to gender and found that the sample comprised 135 men (55.32%) and 109 women (44.67%). As seen in Table 2, there are some differences between the two groups in each dimension of the sense of online community.

**Table 2:** Descriptive statistics of the variables.

**Variable**	**Total (*****n*** **=** **244)**				**Male (*****n*** **=** **135)**				**Female (*****n*** **=** **109)**			
	**Min**	**Max**	**Mean**	**SD**.	**Min**	**Max**	**Mean**	**SD**.	**Min**	**Max**	**Mean**	**SD**.
Mood	0	1	0.55	0.23	0	1	0.47	0.28	0	1	0.51	0.29
Identity	0	801	55.65	90.43	1	801	61.70	104.92	0	369	48.16	68.09
Trust	22	6,084	747.74	853.50	22	6,084	813.01	949.62	49	4,604	666.91	712.92
Informational	0	421	27.80	49.58	0	256	25.84	44.27	0	421	30.23	55.56
Emotional	0	43	3.36	6.67	0	43	3.11	7.02	0	35	3.67	6.22
Socializing	0	71	27.92	32.67	0	279	28.04	33.87	0	193	27.78	31.27

Table 3 presents the correlation matrix for all of the measured variables, containing the dependent variable, the independent variables and the moderating variable. As seen from the correlation matrix, the correlations among all variables are not high, indicating no problem of high multicollinearity.

**Table 3:** Correlation for the variables.

**Variable**	**(1)**	**(2)**	**(3)**	**(4)**	**(5)**	**(6)**	**(7)**
(1) Identity	1.000						
(2) Trust	0.075^*^	1.000					
(3) Informational	0.274^*^	0.033^*^	1.000				
(4) Emotional	0.126^*^	0.028^*^	0.014^*^	1.000			
(5) Socializing	0.325^*^	0.025^*^	0.203^*^	0.146^*^	1.000		
(6) Gender	0.029^*^	0.060^*^	−0.009^*^	−0.005	−0.013^*^	1.000	
(7) Mood	0.028^*^	0.183^*^	0.005	−0.010^*^	−0.154^*^	−0.067^*^	1.000

* *Correlation is significant at the 0.01 level (2-tailed)*.

We find that there is a significantly positive correlation relationship among all the independent variables. For example, the two types of the sense of belonging (*Identity* and *Trust*) are all positively correlated with the three types of the sense of support (*Informational, Emotional* and *Socializing*), indicating that those users who have a strong sense of identity and a high level of trust will be more popular in the community, and they are likely to receive more informational and emotional support, and meanwhile develop stronger social interaction ties with other community members.

In the correlation between dependent variable and independent variables, the findings in Table 3 indicate that all independent variables except *Informational* are significantly related to the dependent variable *Mood*. The preliminary result provides initial support for our hypotheses.

### Hypotheses Tests

We constructed two panel data models to test all of the hypotheses. In the first stage, Model 1 regressed the dependent variable *Mood* on the five independent variables *Identity, Trust, Informational, Emotional* and *Socializing*, to test the effects of different online community activities on depression improvements. In the second stage, the moderator variable *Gender* and the interaction terms between *Gender* and the five independent variables were added to Model 1 to test the moderating effect of gender on the relationship of online community participation and depression improvements.

Table 4 presents the result of panel data regression analysis. We can see that in model 1, the *F*-value of 2979.791 is significant (*p* < 0.01), indicating that the overall fit of model 1 is statistically significant at this level. The result in model 1 shows that the five independent variables are all significant predictors of improvement in mood. The detailed analyses are as follows.

**Table 4:** Panel data regression analysis.

	**Model 1**		**Model 2**	
	**β**	***t***	**β**	***t***
**Main effects**				
Identity	0.154^***^	40.83	0.166^***^	27.83
Trust	0.110^***^	82.93	0.118^***^	58.36
Informational	0.011^**^	2.40	0.006	0.84
Emotional	0.058^***^	4.90	0.061^***^	3.57
Socializing	−0.118^***^	−70.36	−0.122^***^	−48.85
_cons	0.428^***^	80.22	0.450^***^	58.93
**Moderating effects**				
Gender			−0.058^***^	−3.94
Identity^*^Gender			−0.029^^***^^	−2.60
Trust^*^Gender			−0.020^^***^^	−5.15
Informational^*^Gender			0.013	0.94
Emotional^*^Gender			−0.009	−0.26
Socializing^*^Gender			0.010^**^	2.00
**Model evaluation**				
Number of observations	103402		103402	
R-squared	0.131		0.140	
*F*-value	2979.791^^***^^		1361.424^^***^^	

*** p < 0.01,

** *p < 0.05*.

First, the coefficient of the independent variable *Identity* is significantly positive (B = 0.154, *p* < 0.01), indicating that the sense of identity has a positive effect on depression. Thus, Hypothesis 1 is supported. Second, the coefficient of the independent variable *Trust* is significantly positive (B = 0.110, *p* < 0.01), indicating that trust has a positive effect on depression. Thus, Hypothesis 2 is supported. Third, the coefficient of the independent variable *Informational* is significantly positive (B = 0.011, *p* < 0.05), indicating that informational support has a positive effect on depression. Thus, Hypothesis 3 is supported. Fourth, the coefficient of the independent variable *Emotional* is significantly positive (B = 0.058, *p* < 0.01), indicating that emotional support has a positive effect on depression. Thus, Hypothesis 4 is supported. Finally, the coefficient of the independent variable *Socializing* is significantly negative (B = −0.118, *p* < 0.01), indicating that socializing support has a negative effect on depression. Thus, Hypothesis 5 is not supported.

We further analyze the regression results of model 2 in Table 4 to examine whether there are differences concerning the effect of online community participation on depression for male and female users. The F-value of 1361.424 is significant (*p* < 0.01), indicating that the overall fit of model 2 is statistically significant at this level. The results show that the coefficient of *Gender* is significantly negative (B = −0.058, *p* < 0.01), indicating that male users involved in online community have less significant improvement on depression than female users. The detailed analyses of the moderating effects of gender are as follows.

First, the coefficient of interaction between *Identity* and *Gender* is significantly negative (B = −0.029, *p* < 0.01), with the opposite sign to the coefficient of *Identity*, indicating that the positive influence of the sense of identity on depression improvement is weaker for male users than for female users. Hence, H6-a is supported. Second, the coefficient of interaction between *Trust* and *Gender* is significantly negative (B = −0.020, *p* < 0.01), with the opposite sign to the coefficient of *Trust*, indicating that the positive influence of trust on depression improvement is weaker for male users than for female users. Hence, H6-b is supported. Third, the coefficient of interaction between *Informational* and *Gender* is not significant (B = 0.013, *p* > 0.1), indicating that there is no significant gender difference in the effect of informational support on depression. Hence, H6-c is not supported. Fourth, the coefficient of interaction between *Emotional* and *Gender* is not significant (B = −0.009, *p* > 0.1), indicating that there is no significant gender difference in the effect of emotional support on depression. Hence, H6-d is not supported. Finally, the coefficient of interaction between *Socializing* and *Gender* is significantly positive (B = 0.010, *p* < 0.05), with the opposite sign to the coefficient of *Socializing*, indicating that the negative influence of socializing support on depression improvement is weaker for male users than for female users. In other words, socializing support in online depression communities has a more negative effect on depression for female users than for male users. Hence, H6-e is not supported.

We further divided the full sample into subsamples according to gender. Table 5 shows the regression results for men and women users separately. We found that the coefficients of all the five independent variables in Models 3 and 4 are all significant and have the same signs as the coefficients in model 1 except the variable *Informational* in Model 4. The results further support the tests of H1–H5.

**Table 5:** Regression analysis for two subgroups according to gender.

	**Model 1 (total)**		**Model 3 (men)**		**Model 4 (women)**	
	**β**	***t***	**β**	***t***	**β**	***t***
**Main effects**						
Identity	0.154^***^	40.83	0.146^**^	31.06	0.166^***^	26.73
Trust	0.110^***^	82.93	0.105^***^	61.35	0.118^***^	56.06
Informational	0.011^**^	2.40	0.014^**^	2.32	0.006	0.80
Emotional	0.058^***^	4.90	0.054^***^	3.42	0.061^***^	3.43
Socializing	−0.118^***^	−70.36	−0.115^***^	−52.58	−0.122^***^	−46.94
_cons	0.428^***^	80.22	0.409^***^	60.66	0.450^***^	57.71
**Model evaluation**						
Number of obs.	103,402		58,001		45,401	
R-squared	0.131		0.142		0.130	
*F*-value	2979.791^***^		1687.424^***^		1306.093^***^	

*** p < 0.01,

** *p < 0.05*.

In addition, we found that the coefficients of *Identity* and *Trust* in Model 4 are greater than are those in Model 3 (0.166>0.146, 0.118>0.105), showing that the sense of identity and trust have a more positive effect on depression improvement for female users than for male users. The results further support the tests of H6a and H6b. We also made a comparative analysis of the three types of the sense of support in model 3 and 4 and found that the coefficient of *Informational* in model 4 is not significant (B = 0.006, *p* > 0.1), the coefficient of *Emotional* in model 3 and 4 are close to each other (0.054 and 0.061), and the the coefficient of *Socializing* in model 4 is less than that in Model 3 (−0.122 < -0.115). We can conclude that there might be no significant difference in the influence of informational and emotional support on depression improvement between men and women user groups, whereas socializing support will have a more negative effect on depression for female users than for male users. The results further support the tests of H6c–H6e.

## Discussion

First of all, the results of this study show that the usage of online health community is, on the whole, beneficial for users with depressed symptoms to improve their depression outcomes.

The findings confirm the proposed arguments that online interactions in online depression communities can create a sense of community among users, and help them diminish the feelings of isolation and lack of support, buffer the negative impact of depressed mood, and promote positive psychosocial outcomes.

Secondly, we categorized the sense of community into two dimensions: the sense of belonging and of support. We found that both of the two key facets of the sense of belonging: a sense of shared identity and trust have positive effects on depression. A reasonable explanation is that a sense of shared identity and trust are very helpful for users affected by depressed symptoms to enhance mutual understanding and view themselves as a part of the community. They are more willing to engage in online communities to freely exchange information and opinions, which makes them gain high self-satisfaction and thus improve their mental well-being.

In addition, we found that three types of support have different effects on depression. Informational support has positive effect on depression. One reasonable explanation for this is that when users gain much valuable information and knowledge about depression, they are more capable of managing their depressive symptoms. The result is in accordance with some previous researches, proposing that health information provided by social media could facilitate health self-management. Emotional support has a positive effect on depression. It is well-understood that the encouragement and support provided by community members can help eliminate fears and anxiety, as well as strengthen users' confidence in fighting depression. The findings support the idea that users engaged in online health communities could change their emotional state in a positive direction by encouraging each other. Socializing support has a negative effect on depression, which is contrary to the prediction of the hypothesis 5. Some studies based on emotional contagion theory provided a possible explanation about the result. They suggested that, in the context of online health community, negative emotion expressed by some users will infect other group members and make their symptoms of depression worse. Another possible explanation is that when users affected by depressed symptoms spend too much time engaging in online community to maintain social ties with their peers online, they will have not enough time to develop intimate relationships with their family members and other friends in the real world. The argument is supported by Yeh et al. (71) proposing the idea that lower actual social support and higher virtual social support were associated with higher depressive symptoms.

Finally, we further focused on the moderating role of gender and obtained valuable findings. The results show that both of the two key facets of the sense of belonging: the sense of shared identity and trust have more positive effects on depression for female users than male users. The findings indicated support for social role theory suggesting that women are more communal and more likely to develop a strong sense of shared identity and trust with other community members, which make women benefit more than men from online interactions.

We also note that there is no significant gender difference in the effect of informational and emotional support on depression. The results did not support our hypotheses that informational support has a more positive effect on depression for men than women, while emotional support has a more positive effect on depression for women than men. There have been some possible explanations for the findings. For example, women are generally considered to have less power and status than men in most societies, so many women users might value more informational support and take full advantage of online community to acquire available information about depression, especially for the older women with low socioeconomic resources. Besides, contrary to the assertion that women need more emotional support, some studies take several contextual factors into consideration and reach different conclusions. For example, studies found that men living without a spouse in the household are more likely to lack emotional support and are more vulnerable for depression than women in the same situation. Accordingly, emotional support has probably a more positive effect on depression for men living without a spouse. It is notable, however, socializing support has a more negative effect on depression for female users than for male users. One possible reason is that when women are more concerned about socializing with peers to form a larger social network, they inevitably experience more negative impact on their social relationships than men. Some studies found that women are most likely to be affected by negative life events from network members (traffic accidents, divorce, death of partners, or children, etc.) and become more depressed (16). The results also support the idea that participation in social networks might be more harmful than helpful and may paradoxically increase levels of mental illness symptoms, especially if such connections entail role strain associated with obligations to provide socializing support to others.

## Conclusion

### Implications

Online depression communities have become increasingly recognized as promising platforms for communication about depression. While many studies focus on the benefits of online depression communities, some scholars express the opposite opinion that long-term online interactions with major patients may worsen the symptoms of depression. It is still an issue of debate concerning whether online interaction is beneficial or harmful to users with depressed symptoms and, if so, to what extent. In this paper, we adopted an empirical approach to investigate whether online community participation help improve depression outcomes and determine which types of usage behaviors have positive or negative effects on depression.

The finding of this study has implications for research in online depression community. Although some previous studies were conducted to analyze the needs of patients by using topic detection and sentiment analysis of posts in online depression community, few have studies focus on understanding the mechanisms of how online community participation is related to the improvements on depression symptoms. This study attempts to apply the concept of sense of community to the context of online depression community, and suggests that motivation for users' participation in online communities may be a desire to develop a sense of belonging to depression communities and a sense of support from other depression members. We develop a new theoretical model proposing that two dimensions of the sense of belonging (sense of identity and trust) and three dimensions of the sense of support (informational, emotional, and Socializing) have significant effects on depression improvement. Furthermore, our research extends the theoretical work by considering gender difference in online community participation and its effect on the development of depression. The empirical results reveal that some of the hypotheses were supported whereas others were not.

The finding also has some important practical implications. First, the results reveal that the sense of identity and trust with community members have positive effects on depression. Thus, the managers should encourage users to actively participate in the online community to increase the sense of identity and trust. Second, we found that informational and emotional support have positive effects on depression, but socializing support has negative effects on depression. Thus, managers may employ different policies for different types of support. On the one hand, managers should provide more opportunities for users affected by depressed symptoms to promote online interpersonal communication, encourage them to contribute their knowledge and experience and show sympathy and emotional support for peers. On the other hand, managers must be aware of the negative effects of socializing support and enact restriction policies against excessive use of online community to avoid being harmed by other members' negative emotions. Third, managers must be aware of gender difference in online community participation and could consider to provide differentiated services for male and female users, respectively. For example, we can conclude that female users have a stronger sense of identity and trust with other members to make them benefit more than men, but in the meanwhile they are most likely to be affected by negative life events from network members and become more depressed. Thus, managers might try to give female users more encouragement to motivate them to share their experiences with others and increase their self-disclosure, while discouraging female users to develop too close relationship with community members to avoid making their conditions worse.

### Limitations and Future

This study has some limitations. First, we consider that some other demographic factors, including age, ethnicity, region, etc., may play important roles in the improvements on depressed symptoms for online users. However, considering that it is difficult to obtain empirical data on demographics in our sample due to ethical or legal concerns, it is temporarily impossible to study the other demographic factors. We will consider the issue in further studies. Second, we only identify five factors that may have significant effect on depression. The assumed model is somewhat simplified. For instance, it does not take into account the types of informational support (symptoms, medication, treatment, etc.) and emotional support (sympathy, agreement, encouragement, etc.). A more detailed consideration is needed in future. Third, the study results are only applicable to long-term participants. The vast majority of short-term participants will not visit the online community again after posting only a few posts. It is difficult using our method to study the influence of participation behavior on the improvement of depressed symptoms for these short-term participants because we have no enough empirical data to test the proposed hypotheses. Finally, the measure of some variables used in the study may not be accurate enough. For example, we used the sentiment scores of posts written by users as the measure of user depressive symptom. The measure may not be sensitive enough to detect depressive symptom. However, users may participate in other external social media, which we could not collect such data. We consider collecting more accurate normative information about depression through surveys and questionnaires to make the model more realistic in further studies.

## Data Availability Statement

The original contributions presented in the study are included in the article/supplementary material, further inquiries can be directed to the corresponding author/s.

## Ethics Statement

Ethical review and approval was not required for the study on human participants in accordance with the local legislation and institutional requirements. Written informed consent for participation was not required for this study in accordance with the national legislation and the institutional requirements.

## Author Contributions

YL: conceptualization, project administration, and funding acquisition. TP and JL: methodology and data curation. YL and TP: formal analysis and writing—original draft preparation. JW: investigation and supervision. YL and JW: writing—review and editing. TP: visualization. All authors contributed to the article and approved the submitted version.

## Conflict of Interest

The authors declare that the research was conducted in the absence of any commercial or financial relationships that could be construed as a potential conflict of interest.

## Appendix 1

**Table A1:** Some Examples with sentiment scores.

**Postid**	**Post content**	**Sentiment score**
1048058	小组里的每一个人都很棒！交流，分享，成长，关爱自己，传递阳光。感谢你们每一位！ (in Chinese) Everyone is great in the group! Thank you all for Communicate, share, growth, care for yourself, pass the sunshine. (in English)	0.999313
128317	感动于楼主的坚持，也感动于精灵的建议，精灵说得真好！ (in Chinese) It is moved by the persistence of author and the suggestion of the elf. Elf's words are very good! (in English)	0.998895
428510	这几天感觉真的很好！喜普妙和舒思效果真不错！ (in Chinese) I feel really good recently! The effect of Xipumiao and Shusi is really good! (in English)	0.998879
282935	楼下花园里的桂花开了。我希望不要下雨下雪我希望天天都能刮大风。 (in Chinese) The osmanthus are blooming in the downstair garden. I hope it will be windy rather than rain and snow every day. (in English)	0.576289
164071	失眠和抑郁一块治疗。 (in Chinese) Insomnia and depression are treated together. (in English)	0.539584
399859	气死了。真是无语，自误的不少呢。 (in Chinese) It is so angry. There's nothing to say and a lot of self-misunderstood. (in English)	0.19551
781171	爱别离，求不得—苦啊！ (in Chinese) It is painful for the faded love. (in English)	0.195285
